# The Fall of Sleep K-Complex in Alzheimer Disease

**DOI:** 10.1038/srep39688

**Published:** 2017-01-03

**Authors:** Luigi De Gennaro, Maurizio Gorgoni, Flaminia Reda, Giulia Lauri, Ilaria Truglia, Susanna Cordone, Serena Scarpelli, Anastasia Mangiaruga, Aurora D’atri, Giordano Lacidogna, Michele Ferrara, Camillo Marra, Paolo Maria Rossini

**Affiliations:** 1 Department of Psychology, “Sapienza” University of Rome, Rome, Italy; 2 Institute of Neurology, Catholic University of The Sacred Heart, Rome, Italy; 3 Department of Biotechnological and Applied Clinical Science, University of L’Aquila, L’Aquila, Italy; 4 IRCCS San Raffaele Pisana, Rome, Italy

## Abstract

Although a slowing of electroencephalographic (EEG) activity during wakefulness and –to some extent- sleep of Alzheimer disease (AD) patients (i.e., *increased* slow-frequency activity) was documented, recent findings in healthy elderly show a *decreased* 0.6–1 Hz slow wave activity (SWA) during NREM, which was associated to β-amyloid deposition and impaired hippocampal memory consolidation. We hypothesize that the apparent contradiction may be explained by the partial overlap between 0.6–1 Hz EEG activity and K-Complex (KC). According to this view, we studied both frontal KCs and SWA in 20 AD patients and 20 healthy age-matched controls (HC) during nightly sleep, under the hypothesis that KCs better discriminate patients from healthy elderly than ≤1 Hz SWA. A drastic decrease of KC density during stage 2 NREM was found in AD compared to HC. Patients show more than 40% reduction of the KC density, allowing a correct classification of 80%. On the other hand, ≤1 Hz SWA of AD patients is slightly (not significantly) higher in most cortical areas compared to HC. Although no significant changes of ≤1 Hz SWA are detectable over frontal areas in AD, KC density decreases over the same location, and its decrease is related to the cognitive decline.

Alzheimer’s disease (AD) is characterized by several sleep alterations accompanying cognitive decline: the neural degeneration can induce *per se* alterations of sleep pattern and quality, which in turn may provoke a worsening of the cognitive decline, i.e. by the impairment of sleep-dependent memory consolidation processes^1^. The role of sleep as a factor which increases the rate of β-amyloid clearance^2^ raised a further interest in investigating specific sleep markers of AD.

The relatively consolidated electroencephalographic (EEG) signature of AD patients is represented by the phenomenon of “slowing”, a topographically diffuse slow-wave and theta activity characterizing wake and –to some extent- sleep EEG^1^,^3^,^4^.

## Slow-wave activity (SWA)

The slowing of the EEG during wakefulness mostly affects frontotemporal areas^5^. During REM sleep, it appears as an increase of delta and theta power, which is larger in the temporoparietal and frontal regions^6^, while in NREM it is evidenced by a parietal decrease of sigma power^7^ and an increase of high (1.6–3.6 Hz) delta power^8^.

Apparently contradictory, a recent study showed in healthy subjects that the β-amyloid deposition is associated with an *impaired* frontal generation of NREM slow-wave activity (0.6–1 Hz) that, in turn, predicts hindered hippocampal memory consolidation^9^. The same group also demonstrated that age-related NREM slow-wave activity mediates the relation between prefrontal grey matter atrophy and impaired long-term memory and reduced hippocampal-prefrontal functional connectivity^10^. We hypothesize that these findings do not necessarily contradict the phenomenon of EEG slowing in AD patients, and that the <1 Hz slow wave activity (SWA) may not correspond to delta waves but to another widespread event which occurs in NREM sleep, the K-complex (KC)^11^.

## K-complexes (KCs)

KC is one of the hallmarks of NREM sleep and represents the EEG graphoelement with highest amplitude during normal sleep. KC was described more than 70 years ago and since then its physiology was evaluated in hundreds of studies (for a review^12^,). It is characterized by a short surface-positive transient followed by a slower, larger surface-negative complex with peaks at 350 and 550 ms, then a final positivity peaking near 900 ms, all of them being generated in widespread cortical locations^11^. Several studies in cats and humans by Mircea Steriade demonstrated that KCs have a <1 Hz frequency^13^,^14^,^15^. KCs can be spontaneous, but sensory stimulation increases their probability in the ascending slopes of the sleep cycles to a higher degree than in the descending ones^12^. There is a close connection between the formation of KCs and phasic sensory activation^12^. At the same time, a relation between KC formation and the measure of sleep synchronization was observed^16^. The frequency of KCs decrease from cycle to cycle, parallel with the decrease in the depth of sleep^17^. It is assumed that the EEG phenomena of KCs during sleep arise under the effect of continuous sensory activation, hence they correspond to non specific evoked potential elements; therefore the KC can be regarded as a building stone of slow-wave sleep^16^, and has been considered a sort of ‘guardian’ for sleep protection.

Converging evidence in animals and humans points to a cortical origin of the KCs^11^,^15^,^18^,^19^, while the thalamus seems to have a role in mediating the cortically generated KCs^15^,^20^. KC is characterized by a frontal predominance^12^,^21^,^22^, and it shows a decrease in individual with alcohol use disorder, with a predominant effect at frontal brain regions^23^. Normal aging is associated with a decrease of spontaneous^24^,^25^ and evoked KCs^26^,^27^,^28^. This decrease is again more pronounced over the frontal cortex^28^. Preliminary observations also suggested that AD patients have a further reduction of spontaneous KCs^29^,^30^,^31^.

These converging pieces of evidence legitimate the hypothesis that the relation between decreased frontal 0.6–1 Hz SWA and β-amyloid deposition, mechanistically contributing to the hippocampus-dependent cognitive decline in the elderly^9^, might be – at least in part- explained by a reduced generation of spontaneous KCs. Therefore we hypothesize that: (A) KCs better discriminate AD patients from healthy elderly than slower (0.6–1 Hz) SWA, and (B) the reduced generation of spontaneous KCs in AD patients should correlate with the extent of cognitive decline, as measured by the Mini Mental State Examination (MMSE) scores.

## Results

### Demographic and clinical characteristics

Results of the t-tests performed on demographic and clinical characteristics of AD and healthy elderly control subjects (HC) are reported in Table 1. A significant between-groups difference was observed for MMSE scores with higher scores in HC than in AD, and for State Trait Anxiety Index (STAI Y1 and STAI Y-2) with lower scores in HC than in AD. No significant difference was observed for age, education, Hamilton Depression Rating Scale (HDRS) and Pittsburgh Sleep Quality Index (PSQI).

### Sleep measures

Table 2 reports the results of the t-tests performed on polysomnographic (PSG) measures. A significant difference was found for slow wave sleep (SWS), with a higher percentage of SWS in HC compared to AD. Moreover, a significant increase of Stage 1 and Stage 2 latency in AD compared to HC was observed.

**Figure 1:**
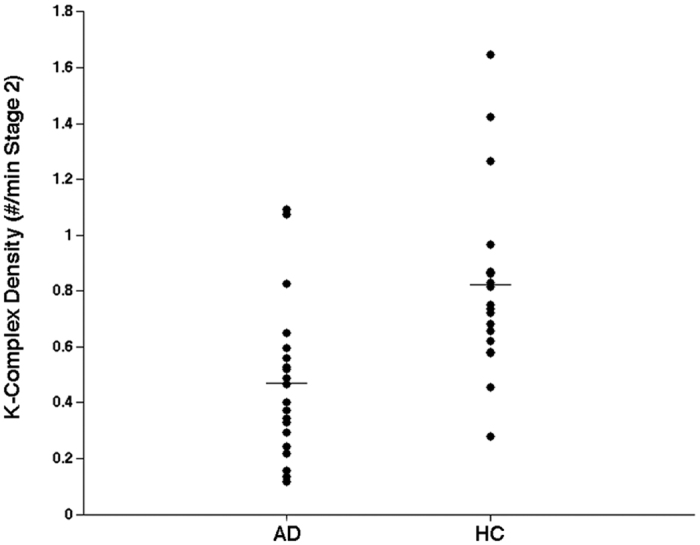
Absolute values and mean of K-Complex (KC) density of Alzheimer’s Disease (AD) patients and healthy controls (HC) at Fz cortical derivation.

**Figure 2:**
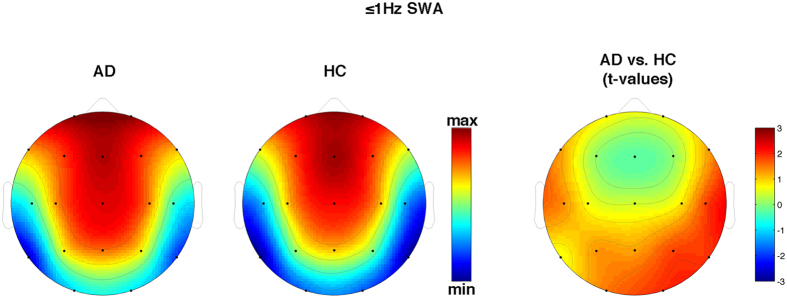
Topographical scalp maps of 0.6–1 Hz (≤1 Hz) slow-wave activity (SWA) in AD patients and HC. The maps are based on the 19 derivations of the 10–20 system (electrodes positions indicated by black dots). Values are color-coded and plotted at the corresponding position on the planar projection of the hemispheric scalp model. Values between electrodes were interpolated (biharmonic spline interpolation). Values are expressed in terms of log values of ≤1 Hz SWA during NREM sleep of the two groups (left side) and values of the between group comparisons (t-test values on the right side).

**Figure 3:**
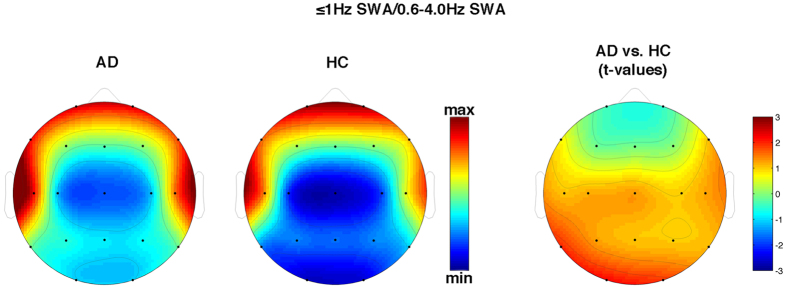
Topographical scalp maps of the ratio between 0.6–1 Hz (≤1 Hz) activity and the whole (0.6–4 Hz) slow-wave activity (SWA) in AD patients and HC. The maps are based on the 19 derivations of the 10–20 system (electrodes positions indicated by black dots). Values are color-coded and plotted at the corresponding position on the planar projection of the hemispheric scalp model. Values between electrodes were interpolated (biharmonic spline interpolation). Values are expressed in terms of ratio between 0.6–1/0.6–4 Hz EEG activity during NREM sleep of the two groups (left side) and values of the between group comparisons (t-test values on the right side).

As hypothesized, we found a strong reduction of KC density in AD compared to HC (t_1,38_ = 3.70, p = 0.0007) with a 42.7% decrease (Fig. 1). On the other hand, the between-group comparisons of ≤1 Hz SWA never reached significance at any scalp location (p > 0.10), although the general pattern suggests a relative slowing of EEG in AD, except for frontal sites (Fig. 2). This general pattern is confirmed when considering the proportion of ≤1 Hz SWA over the whole SWA (Fig. 3).

The discriminant analyses revealed KC density as the best classifier (F_1,37_ = 15.11; p = 0.0004), allowing a correct classification of 80%.

### The relation between KCs and cognitive impairment

The multiple regression predicting MMSE scores on the basis of KC density and ≤1 Hz SWA was significant (r_multiple_ = 0.49; p = 0.02). The only significant predictor was KC density (β = 0.54; partial r = 0.50). Interestingly, the non significant partial correlation between ≤1 Hz SWA and MMSE was negative (β = −0.20; partial r = −0.21). As expected, KC density and ≤1 Hz SWA were interrelated (r = 0.38; p = 0.02).

### Control analysis on KCs regional differences

**Figure 4:**
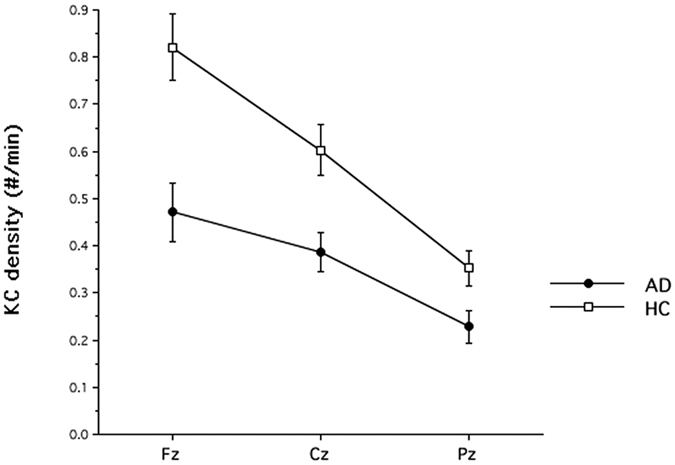
Means and standard errors of KC density in Alzheimer’s Disease (AD) patients and healthy controls (HC) recorded over midline derivations (Fz, Cz, and Pz).

According to the main hypothesis of our study and to the evidence in normal aging^24^, the fall of spontaneous KCs should be more pronounced over the frontal cortex. To this aim, we also scored KCs over the available midline derivations (i.e., Cz and Pz). The mixed-design Group (HC, AD) × Derivation (Fz, Cz, Pz) analysis of variance yielded a significant main effect for the Group factor (F_1,38_ = 13.42; p = 0.0007), with a lower KC density in AD (X = 0.36; SE = 0.04) than in HC (X = 0.59; SE = 0.05). The main effect for the Derivation factor was also significant (F_2,76_ = 61.67; p < 0.00000001), with a significant linear decrease from anterior to posterior sites (linear trend: F_1,38_ = 74.24; p < 0.00000001). Finally, the Group x Derivation interaction (Fig. 4) was significant (F_2,76_ = 6.24; p = 0.003), and it shows that the reduction in frontal KC activity was greater than that at the other brain regions (Fz = 42.7%, Cz = 36.0%; Pz = 35.4%).

## Discussion

Our results show a striking decrease of frontal spontaneous KC density during NREM sleep in AD patients compared to HC. The reduction of KC density over the frontal area of AD patients exceeds 40%. On the other hand, SWA on the same frontal site is not significantly different between groups. In contrast to SWA, KC density was also positively related with MMSE scores.

In agreement with previous PSG studies^1^, sleep of our AD patients is characterized by a decrease of SWS and a lengthening of sleep latency. However, the quantitative EEG analysis points to a more articulated regional pattern. The general tendency of AD patients to present a higher ≤1 Hz SWA compared to HC appears counterbalanced over the frontal areas by a relative decrease of KCs. In fact, our control analysis shows that the reduction of KCs is larger over the frontal site, and it diminishes progressively along the antero-posterior axis. Therefore, the overall tendency of SWA to increase in AD is obscured on the cortical area where the drop of KCs is more pronounced. According to this interpretation, the empirical finding in healthy elderly people of an association between the β-amyloid burden and SWA^9^,^10^ could be hardly replicated in AD patients, due to the dissociation between increased SWA and decreased KC density.

A similar dissociation was reported during SWS in demented patients compared to normal elderly^8^. Albeit the relatively small sample size (but still the largest in the literature) and the heterogeneity of the dementia group, this study reported a decreased amplitude and incidence of slow delta waves (<1.6 Hz) and an increased incidence of fast delta waves (2–3.6 Hz). Since KCs mostly correspond to slow delta frequency events and some evidence suggests that they may be considered as single instances of delta EEG activity that predominates in SWS^16^,^18^,^32^, the observation by Bonanni and coworkers^8^ is coherent with the decreased incidence of KCs in dementia.

In our opinion, the dissociation between KCs and SWA depends on the cortical origin of the former. The cortical origin of KCs is supported by several studies^11^,^15^,^19^, and their generation seems associated with cortical slow (<1 Hz) oscillations^15^. Since glial cells seem to have a significant role in producing slow oscillations^33^, glial alterations were proposed as possible neural basis for the impaired cortical ability to produce KCs^34^. Alternatively, AD-related damage at the level of the cholinergic basal forebrain^35^,^36^ may account for KCs alterations^34^, in line with a model of KCs generation proposed by Colrain and Crowley^37^, which involves the connection between the brainstem reticular activating system, the basal forebrain and the cortex. In any case, four different phenomena are at work in AD patients: (1) the drop of KCs, more pronounced over the frontal areas; (2) a lack of differences over the same frontal areas in ≤1 Hz EEG activity; (3) a general slowing of EEG activity (not significant probably due to the relatively small sample size); and (4) the decrease of visually scored SWS (paralleled by a lengthening of sleep latency). While the last effect is likely an expression of a worse sleep quality, the others have a different origin.

Beyond extending the preliminary evidence of KC alterations in AD patients^31^,^34^, we also show that the drastic decrease of KCs is related to the cognitive impairment. This relation may give some hints about the functional significance of the KC density decrease in AD patients. Results from Cash and co-workers^11^ show that KCs are generated in widespread cortical areas and are accompanied by a decrease in cortical activity, representing isolated cortical down-states. Sleep-related cortical down-states are thought to be associated with a reduction of synaptic strength that should counterbalance the wake-related synaptic enhancement, and this mechanism seems to be strongly related with memory processes and brain cellular energy regulation^38^. In this view, the reduced ability to produce KCs in AD patients may be associated to a disruption of sleep-related memory processes, contributing to the cognitive deterioration in these patients. The relation mutually linking impaired long-term memory and reduced hippocampal-prefrontal functional connectivity and age-related prefrontal grey matter atrophy and lower slow-wave activity, respectively^9^,^10^, goes in the same direction. Hence, our finding further supports the notion of a bidirectional influence between sleep alterations and cognitive deterioration in AD: the neurodegenerative process affect the hypnic profile and pattern (i.e. reduction of KCs density), which in turn provokes a further deterioration of the cognitive status presumably by negatively impacting on synaptic plasticity and memory consolidation processes.

From a clinical point of view, the present findings provide further evidence about the relation between sleep alteration and cognitive functioning in AD, endorsing sleep improvement as a possible strategy to reduce the AD-related cognitive deterioration process and disease progression rate. To this aim, recent studies enhancing slow oscillations in humans by means of electrical^39^,^40^,^41^, transcranial magnetic^42^ and acoustic^43^,^44^,^45^,^46^,^47^ stimulation appear promising. Notably, the boost of slow oscillation during sleep was also associated with beneficial effects on memory processes^39^,^40^,^41^,^43^.

### Limitations to the study

The main limitation of the present study is that our results intrinsically do not allow establishing any causal relation among the several phenomena observed in NREM sleep of AD patients (i.e., the frontal fall of KCs, the absence of differences in the 0.6–1 Hz in the frontal region, the general -not significant- slowing of EEG activity, and the decrease of SWS).

As a second limitation, it must be considered that the beginning of the sleep EEG recordings was fixed at a specific bedtime (23.00) for all subjects. Given the observation that AD patients often exhibit circadian misalignment, it could be argued that a fixed bedtime may have influenced our findings, since we compare AD patients with a group of HC theoretically circadian aligned. However, it is worth noting that the evaluation of a regular sleep-wake cycle was one of the inclusion criteria for the present study (see the ‘Inclusion and exclusion criteria’ section), and this may partially face the issue of a possible circadian bias.

Finally, our study does not evaluate the CAP A1 phase^48^. This is a phasic slow wave event which starts with KC-like huge biphasic slow components. It consists of periods of KC and delta bursts, polyphasic bursts, vertex sharp transients, with <20% of EEG desynchrony. Part of this phasic event may fit into 0.6–1 Hz range, and it would be interesting, in the future, to specifically investigate this issue.

## Conclusions

Sleep may represent a still unexplored, but privileged window on the neurophysiological changes of AD. Our finding suggests that the association in healthy older people between impaired long-term memory, age-related prefrontal grey matter atrophy, β-amyloid plaques burden, and slow-wave activity in the EEG of NREM sleep^9^ cannot be extended to AD patients. The hypothesized increase of ≤1 Hz EEG activity characterizing SWA during NREM sleep is masked by a concomitant (and larger) change of opposite sign. KC, as a phasic slow wave of NREM sleep, is indeed significantly less frequent over frontal areas in AD, and its decrease is related to the cognitive decline as measured by MMSE scores. This also confirms other studies pointing to an involvement of frontal areas in the neurodegenerative process^49^. Future studies should directly investigate the neural basis and the functional role of the AD-related KC decrease by assessing its relation with grey matter atrophy and the deposition of β-amyloid. Above all, these relations should be evaluated by prospective studies aimed at investigating the longitudinal association between KC density reduction, cognitive decline, and neuroanatomical changes.

## Methods

### Subjects

In the present study, 20 AD patients (7 males and 13 females; mean age = 72.0 yrs.) and 20 HC (12 males and 8 females; mean age = 70.3 yrs.) were recruited. Demographic and clinical characteristics of the recruited sample populations are reported in Table 1. Patients were enrolled for the study just after their diagnosis [8 mild AD (MMSE score = 18–23); 8 moderate AD (MMSE score = 13–17); 4 severe AD (MMSE score <13)]. Patients were selected among the elderly persons referred to the Neuropsychology Unit of the Gemelli Foundation, Catholic University Hospital of Rome. HC were recruited in clubs for retired people. All subjects gave their written informed consent. The study was approved by the Institutional Review Board of the Department of Psychology of the University of Rome “Sapienza” and by the Institutional Ethical Committee of the Catholic University Hospital of Rome, and was conducted in accordance with the Declaration of Helsinki.

### Inclusion and exclusion criteria

All participants underwent cognitive screening by means of the Mini Mental State Examination (MMSE)^50^. Moreover, the State Trait Anxiety Index (STAI-Y1 and STAI-Y2)^51^ and the HDRS^52^ were administered in order to exclude major psychiatric illness.

Neuropsychological investigation for AD patients included a structured clinical evaluation, brain neuroimaging (MRI or CT), and a neuropsychological test battery for the assessment of specific cognitive functions such as memory, attention, executive function, visuo-construction abilities and language. In particular, memory assessment included Rey’s Auditory Verbal Learning (RAVLT)^53^, involving immediate recall (RAVLTir = 14.8; SE = 1.8), delayed recall (RAVLTdr = 0.6; SE = 0.3) and delayed recognition (Accuracy of RAVLTrec = 74%; SE = 4.6%), delayed recall of the Rey figures^54^, delayed recall of a three-word list^55^ and delayed recall of a story^56^,^57^. The functional status was assessed by the Activities of Daily Living (ADL = 5.33; SE = 0.3) and Instrumental Activities of Daily Living (IADL) questionnaire^58^. AD patients were included according to the National Institute on Aging-Alzheimer’s Association workgroups^59^ and DSM-IV criteria.

Common exclusion criteria for all participants were: presence of neurological, psychiatric or vascular disorders, obesity, history of alcoholism, drug abuse and hypnotic intake. HC receiving psychoactive drugs were also excluded. The final enrollment in the study was based on the evaluation of regular sleep-wake cycle and on the absence of self-rated sleep disorders. The presence of other sleep disorders was objectively evaluated by nocturnal sleep recordings. In case of sleep disorder and/or respiratory diseases and obstructive sleep apnea syndrome (OSAS), subjects were excluded by subsequent analyses. Sleep quality and diurnal sleepiness of all participants were assessed by the Italian version of the PSQI^60^, the Epworth Sleepiness Scale (ESS)^61^ and the Karolinska Sleepiness Scale (KSS)^62^.

### Study design and experimental schedule

Participants underwent a complete polysomnographic (PSG) recording of a nocturnal sleep at the Catholic University Hospital of Rome. The sleep-wake history of HC in the day preceding the study was controlled by a sleep diary and -as far as possible- by hospital staff for the AD patients. Naps were not allowed, although compliance was not objectively controlled (i.e., by actigraphic recordings) to have daily naps.

Participants arrived in the sleep laboratory around 20.00 (HC from their home and AD patients from the hospital ward, accompanied by their caregivers). After PSG montage, they were requested to fill in the PSQI, ESS, and KSS. Caregivers were asked when patients were not able to respond. Then, resting EEG was recorded for a 5-min eyes-closed period, followed by a 5-min eyes-open period (data not reported here). Sleep recordings started at 23.00. If not already awake, participants were awakened at 6.00. Before leaving the sleep laboratory, their resting EEG was again recorded (5-min eyes-closed and 5-min eyes-open periods).

### Polysomnographic recordings

A Micromed system plus digital polygraph was used for the PSG recording. EEG signals were acquired with a sampling frequency of 256 Hz and band-pass filtered at 0.53–40 Hz. The 19 unipolar EEG derivations of the international 10–20 system (C3, C4, Cz, F1, F2, F3, F4, F7, F8, Fz, O1, O2, P3, P4, Pz, T3, T4, T5, T6) were recorded from scalp electrodes with average mastoid references (A1 and A2), using Ag/AgCl electrodes. Electrooculogram (EOG) was recorded from electrodes placed about 1 cm from the medial and lateral canthi of the dominant eye. Electrocardiogram (EKG) and submental electromyogram (EMG) were also recorded. Finally, a pulse oximeter was placed on the right index finger with the aim to exclude sleep respiratory disorders. Periodic Limb Movements were not measured by a tibialis EMG recording, but excluded on the basis the PSQI evaluation and their clinical history. Any contamination of EEG recordings by muscle activity was however excluded by subsequent analyses. Impedance was kept below 5 KOhm.

## Data Analysis

### Demographics and clinical characteristics

Age, years of education and clinical characteristics (MMSE, HDRS, STAI Y-1, STAI Y-2 and PSQI scores) of AD and HC were compared by two-tailed t-tests for independent samples. Alpha level was always set at 0.05.

### Sleep measures

Sleep stages of the baseline night were scored visually in 20 seconds epochs, according to standard criteria^63^, excluding ocular and muscle artifacts. The following were considered as dependent variables: (a) stage 1 latency; (b) stage 2 latency; (c) total sleep time (TST), defined as the sum of time spent in stage 1, stage 2, SWS and REM; (d) percentage of each sleep stage (time spent in a sleep stage/TST × 100); (e) wakefulness after sleep onset (WASO), in minutes; (f) number of awakenings; (g) number of arousals; (h) total bed time (TBT); (i) sleep efficiency index (SEI = TST/TBT × 100). An awakening was scored whenever an EEG/EMG activation occurred lasting more than 10 s. Arousals were scored whenever an EMG activation affected the EEG recording for periods shorter than 10 s.

The polysomnographic EEG measures were compared by two-tailed t-tests for independent samples.

### KC detection

Spontaneous KCs were visually identified by a blind scorer during NREM stage 2 sleep on Fz. To score a KC, the following criteria were applied: a nonstationary event with (a) a marked and well-delineated sharp wave initially negative in polarity, immediately followed by a positive polarity component; (b) maximum amplitude at frontocentral derivations; (c) a minimum duration of 0.5 s and a maximum duration of 3 s. According to Crowley and coworkers^28^, no amplitude criterion was applied in the present study, since the previously observed amplitude decrease in older subjects^29^. If multiple KCs appeared in sequence, only the first one was considered^64^. KC density was calculated as the number of KCs divided by NREM stage 2 sleep minutes.

### Measure of slow-wave activity

The polygraphic signals (19 EEG channels, EOG and EMG) were analog to digital converted on-line with a sampling rate of 256 Hz and bandpass filtered at 0.53–40 Hz. Ocular and muscle artefacts were excluded off-line by visual inspection. We calculated power spectra for the SWA of NREM sleep within the 0.6–4 Hz range by a Fast Fourier Transform routine for 4 s periodograms. Aimed to match sleep scoring, power spectra were averaged over 5 consecutive 4-sec epochs to yield a 20-s spectrum. Power spectra were calculated for NREM stages (stage 2 + 3 + 4).

EEG power values for the ≤1 Hz SWA, computed within the 0.6–1 Hz range, were the main dependent measure. Values were log-transformed, colour coded, plotted at the corresponding position on the planar projection of the scalp surface, and interpolated (biharmonic spline) between electrodes. According to Mander and coworkers^9^, ≤1 Hz SWA was also expressed as the ratio between 0.6–1/0.6–4 Hz EEG activity (i.e., ≤1 Hz/SWA).

### Statistical analysis

Group difference in KC density was assessed by means of two-tailed t-tests comparing AD and HC. Preliminary analyses also considered Gender as a between factor, without any significant main effect or interaction involving this factor. For this reason, it was collapsed in the subsequent analyses. Similarly, ≤1 Hz frontal SWA and the proportion of ≤1 Hz SWA/SWA were compared between groups. A discriminant analysis was also performed considering KC density and ≤1 Hz frontal SWA as discriminant measures.

Finally, in order to assess the relationship between KCs and cognitive impairment, a multiple regression was computed between Age, KC density and ≤1 Hz frontal SWA, as independent measures, and MMSE scores, as dependent measure.

## Additional Information

**How to cite this article:** De Gennaro, L. *et al*. The Fall of Sleep K-Complex in Alzheimer Disease. *Sci. Rep.* **7**, 39688; doi: 10.1038/srep39688 (2017).

**Publisher's note:** Springer Nature remains neutral with regard to jurisdictional claims in published maps and institutional affiliations.

## Figures and Tables

**Table 1:** Mean and standard errors (SE) of demographic (age, education) and clinical (MMSE, HDRS, STAI Y-1, STAI Y-2, PSQI) characteristics of Alzheimer disease (AD) patients and healthy elderly controls (HC).

Variables	AD	HC	t_1,38_	p
	Mean (SE)	Mean (SE)		
Age (years)	72 (1.92)	70.35 (1.4)	0.69	0.49
Education (years)	9.65 (1.17)	11.60 (1.12)	−1.20	0.24
MMSE	16.40 (1.05)	28.75 (0.29)	**−11.32**	**<0.0001**
HDRS	10.5 (1.24)	7.45 (1.09)	1.86	0.07
STAI Y-1	39.11 (2.19)	33.4 (1.41)	**2.23**	**0.03**
STAI Y-2	41.22 (2.02)	33.65 (1.92)	**2.71**	**0.01**
PSQI	5.10 (0.61)	6.05 (0.68)	−1.03	0.31

MMSE, Mini Mental State Examination; HDRS, Hamilton Depression Rating Scale; STAI Y-1 and Y-2, State Trait Anxiety Index; PSQI, Pittsburgh Sleep Quality Index.

The results of the statistical comparisons (t and p values) were also reported.

**Table 2:** Mean and standard errors (SE) of the polysomnographic variables of Alzheimer disease (AD) patients and healthy elderly controls (HC). The results of the statistical comparisons (t and p values) are included.

Variables	AD	HC	t_1,38_	p
	Mean (SE)	Mean (SE)		
Stage 1 latency (min)	51.02 (13.43)	21.45 (4.96)	**2.07**	**0.05**
Stage 2 latency (min)	42.88 (13.21)	14.15 (3.79)	**2.09**	**0.04**
Stage 1 (%)	11.59 (2.39)	6.71 (1.04)	1.87	0.07
Stage 2 (%)	74.90 (2.65)	76.76 (1.78)	−0.58	0.56
SWS (%)	0.14 (0.07)	0.95 (0.37)	**−2.11**	**0.04**
REM (%)	13.58 (2.54)	15.93 (1.28)	−0.83	0.41
WASO (min)	86.38 (11.37)	83.86 (8.24)	0.18	0.86
Awakenings (#)	17.25 (3.05)	20.30 (1.81)	−0.86	0.4
Arousals (#)	35.70 (7.41)	32.25 (6.09)	0.36	0.72
TST (min)	260.67 (18.74)	300.12 (14.88)	−1.65	0.11
TBT (min)	389.07 (16.79)	397.18 (11.97)	−0.39	0.70
SEI % (TST/TBT)	66.74 (3.66)	75.23 (2.68)	−1.87	0.07

SWS, slow-wave sleep; REM, rapid eye movement; WASO, wake after sleep onset; TST, total sleep time; TBT, total bed time; SEI, sleep efficiency index.
